# Autoimmunity Panels: Needs and Implementation in the Underdeveloped Regions and how to Approach the Disparities

**DOI:** 10.1002/mdc3.13946

**Published:** 2023-12-27

**Authors:** Kailash Bhatia, Bettina Balint

**Affiliations:** ^1^ Department of Clinical and Movement Neurosciences UCL Queen Square Institute of Neurology University College London London United Kingdom; ^2^ Department of Neurology University Hospital Zurich, University of Zurich Switzerland

**Keywords:** antibodies, movement disorders, testing, diagnosis, treatment, underdeveloped countries, India

## Introduction

Similar to the advances in genetics of Movement disorders (MD), there has also been an explosion in the advances in autoimmune conditions causing movement disorders (AIMD).^1^, ^2^ In genetics, the diagnosis has been based on gene panels, exome sequencing and next‐generation sequencing. Similarly, there have been major advances with regard to the different antibodies which cause movement disorders, and obviously antibody panels are important in diagnosing these conditions. There is an overlap between different antibodies causing one particular form of movement disorders and vice versa. What is crucial and unlike most genetic disorders, most neuronal antibody‐mediated MD conditions are eminently treatable, particularly those with autoantibodies targeting surface antigens, (such as anti‐NMDAR, CASPR2, LGI1 etc.), but there are also implications for the paraneoplastic antibody‐related MD to look out for occult neoplasms and to treat/remove those if possible.^3^, ^4^

Early recognition is important from the treatment perspective and outcomes. It is important to note that the diagnosis of antibody‐related movement disorders is based on serum and CSF analysis. There are several different techniques for assessing the presence of antibodies including cell‐based assays, immunoblots, enzyme‐linked immunosorbent assay (ELISA), and immunohistochemistry on brain tissue.^2^, ^5^ Most of the discoveries of antibodies have been made in western countries. However, even in the West, autoantibody testing is mainly available in larger centers or referral units under national health schemes, but also available privately through some commercial laboratories. However, the tests are generally expensive, and many laboratories offer only few antibodies. Many newly discovered antibodies may be available only in reference laboratories, perhaps where they may have first been discovered.^6^, ^7^, ^8^ Taking the UK as an example, antibody tests are available in major centers such as Oxford, London, Sheffield, Newcastle through the NHS, and these are referral hubs providing services to others. An ERN‐EAN‐collaboration is being mooted to determine the burden of autoimmune neurological disease in Europe and the availability of investigations and therapies, and there is a possibility that some countries, particularly in Eastern Europe, may be poorly provided in this regard.

## What is their Availability In Under‐Developed and Upcoming Regions?

Autoimmune MD are also prevalent in the upcoming regions of the world and are being increasingly recognized. Yet, availability of autoimmune panels is generally limited to the larger metropolises, and often only in the private sector rather than at the National health or government‐sponsored health centers. However, these are slowly but increasingly offering at least the basic panels in the national hospitals of major cities. In India (and probably other Asian countries), autoantibody testing is mostly provided by private laboratories, but some National centers also provide some panels (for example AIIMS, NIMHANS; (personal communication Dr Charu Sankhla, Mumbai and Dr Roopa Rajan, New Delhi).

Rural areas and small towns may be underrepresented in this regard.

## Cost and Timing as an Issue

There is also the issue of costs and, also importantly, the time factor, which can be crucial with regard to early diagnosis in the upcoming countries. This may be creating a two‐tier system where the privileged and big city dwellers may be able to avail of tests (and hence timely treatment where needed) earlier than the others from mofussil areas. The cost of a standard AIMD antibody panel (anti‐NMDAR, LGI1, and CASPR2, AMPAR (GluR1 and GluR2 subunit), GABA_B_R, and DPPX) in Mumbai is £300 (equals approximately 373 USD or 343 EUR) for serum and an additional £300 for CSF testing. If a standard paraneoplastic antibody panel is added (anti‐Hu, Yo, Ri, PNMA2, amphiphysin), an additional £300 is needed for serum and CSF. Hence, the total comes to £900 (personal communication Dr Charu Sankhla), which is rather steep for most in a country like India and southeast Asia. New antibodies in AIMD are constantly appearing on the horizon—recent examples being IgLON5 or Kelch‐like protein 11 antibodies. One also needs to keep in mind that the routine panels as mentioned may miss out on antibodies available in specialized research laboratories, so there is a constant need to update the panel to include at least the more important/ frequent new antibodies. Another issue is that, while awareness of AIMD is becoming more widespread in the neurology MD specialist community, this is less in the general neurologist and possibly even less in the lay physician or general practitioner. Hence, the diagnosis may not be considered in the mofussil areas or may be delayed. Antibody tests are obviously expensive, but one also has to keep in mind that further (often expensive) investigations may be needed. For example, those with paraneoplastic antibodies detected may need investigations for occult neoplasms including further imaging (CT, MRIs, whole body PET imaging) or instrumentation which adds to the costs. Finally, if the diagnosis is confirmed, treatments apart from opportune steroid therapy, IVIG, plasmapheresis or other immunotherapies (which may need to be repeated) can add substantial costs.

## What can be Done to Help or Address these Disparities and Issues?

The strength in most less privileged areas is the clinical expertise. Many AIMD have fairly classic and well‐recognized phenotypes. Widespread education of neurologists (both MD and non‐MD) is necessary for knowing these classic phenotypes. In this context, one of the most important phenotypes is anti‐NMDAR encephalitis. It is imperative that the classic phenotype with the stereotyped movements in an unconscious young patient with recent onset of psychosis, is recognized and taught in medical schools and neurology courses. Its variants, including children who present predominantly with a movement disorder without psychiatric symptoms, or choreo‐ballistic relapses after herpes simplex virus encephalitis being caused by NMDAR‐antibodies also need to be recognized. Other characteristic presentations, such as faciobrachial dystonic seizures due to LGI1 antibodies, or a coarse myoclonus of the legs affecting stance and gait with CASPR2 antibodies, either in isolation or with encephalopathic features, and red‐flags for these conditions such as hyponatremia or cardiac arrhythmias, should be recognized. It is also important to know other classic conditions such as stiff person syndrome and its variants, opsoclonus‐myoclonus syndrome and other paraneoplastic syndromes, many of which feature rapidly progressive cerebellar ataxia. Neurology teaching should highlight these as additional treatable MD on the same page as we have Wilson's disease and dopa‐responsive dystonia as two classic treatable MD conditions.

## Ethical Implications Regarding Treatment in Those Empirically Considered to Have an Autoimmune Disorder and Antibodies of Unknown Significance

One question which comes up often is whether one should consider empirical immune therapies in a given case clinically suspected to have an autoimmune encephalitis (AE), but where it has not been possible to test for antibodies either as they were not available, or the patient was not able to afford it, or some other reason. One can give recommendations applicable generally, but this has to be considered on a case‐by‐case basis. Generally, this limitation has been recognized, and hence clinical criteria for AE have been proposed to facilitate early treatment and avoid delay due to missing antibody test results.^9^ These criteria encompass rapid progression of encephalopathy (memory deficits, psychiatric symptoms, altered mental status), plus at least either a new focal neurological deficit, new‐onset seizures, inflammatory CSF, or MRI findings suggestive of autoimmune encephalitis, in the context of a reasonable exclusion of alternative diagnosis. The need of a “presumptive” diagnosis of autoimmune encephalitis to enable early treatment was also emphasized by Pradhan and colleagues, who also proposed a practical approach useful in their experience in India.^10^ Indeed, it is important to keep in mind mimics of autoimmune encephalitis particularly in view of the side effects of immunosuppression and the possibility of flaring up underlying infections, as these are more common in underdeveloped countries. However, in fairly clear cases—(new onset psychosis, characteristic phenotypes, etc.)—immune therapy should be considered with caution despite not having a clear antibody defined either due to its unavailability or it not being detected in the regular panels. One issue also to mention is that of detecting antibodies of unclear significance (AUS) which may occur and may not necessarily be the cause of the observed condition.^5^

## How Do we Leverage and Influence Administrations about the Needs and Disparities?

Interestingly, there is a big push for genetic testing and funding in both developed but now also underdeveloped countries, even though most genetic disorders cannot be treated apart from very few exceptions. In contrast, many antibody‐related conditions, particularly those with neuronal surface antibodies, are eminently treatable. This recognition has to be made through education of the medical fraternity and also of managers and governing bodies of health institutions and organizations. More funding for public hospitals and organizations needs to be made available for both the investigations and the therapies of immune‐mediated conditions. Antibody‐related conditions such as myasthenia gravis or neuromyelitis optica have become well known in neurology even at basic levels, and neurologists and physicians will almost routinely ask for antibodies and are aware of these disorders which is made clear in most medical curriculums. AIMD such as anti‐NMDAR encephalitis and probably anti‐LGI1‐ and Caspr2‐related disorders deserve the same recognition as they are eminently recognizable and treatable. This needs to be taken up at both at the regional and central levels and officers and administrators made aware of this necessity. Local audits/studies to assess such needs and treatment delivery and outcomes need to be done at regional and National levels to leverage this. Publicity from such forums and publications in relevant journals would be necessary to show that these are treatable disorders, and it is important to recognize them. There is hope that the new generation of MD specialists will be able to take forward this message (Video 1).

**Video 1:** Autoimmunity panels: Needs and implementation in the underdeveloped regions and how to approach the disparities.

## Author Roles

(1) Research project: A. Conception, B. Organization, C. Execution; (2) Statistical Analysis: A. Design, B. Execution, C. Review and Critique; (3) Manuscript Preparation: A. Writing of the first draft, B. Review and Critique.

K.B.: 3A

B.B.: 3B

## Disclosures

**Ethical Compliance Statement:** The authors confirm that the approval of an institutional review board was not required for this work. We confirm that we have read the Journal's position on issues involved in ethical publication and affirm that this work is consistent with those guidelines.

**Funding Sources and Conflicts of Interest:** No specific funding was received for this work. There are no conflicts of interest to report.

**Financial Disclosures for Previous 12 Months:** KPB received speaker honoraria from Ipsen, Merz, MDS; personal compensation for scientific advisory board for Mitsubishi (Neuroderm), Jazz Pharma and Ipsen; receives royalties from the publication of Oxford Specialist Handbook of Parkinson's Disease and Other Movement Disorders (Oxford University Press, 2008), Cambridge Press; and editorial work stipend from MDS for MDCP journal. BB reports they received royalties from Oxford University Press.
